# The CARD9-Associated C-Type Lectin, Mincle, Recognizes La Crosse Virus (LACV) but Plays a Limited Role in Early Antiviral Responses against LACV

**DOI:** 10.3390/v11030303

**Published:** 2019-03-26

**Authors:** João T. Monteiro, Kathleen Schön, Tim Ebbecke, Ralph Goethe, Jürgen Ruland, Wolfgang Baumgärtner, Stefanie C. Becker, Bernd Lepenies

**Affiliations:** 1 Immunology Unit & Research Center for Emerging Infections and Zoonoses, University of Veterinary Medicine Hannover, 30559 Hannover, Germany; joao.monteiro@tiho-hannover.de (J.T.M.); kathleen.schoen@tiho-hannover.de (K.S.); tim.markus.ebbecke@tiho-hannover.de (T.E.); 2 Institute for Parasitology and & Research Center for Emerging Infections and Zoonoses, University of Veterinary Medicine Hannover, 30559 Hannover, Germany; 3 Institute for Microbiology, University of Veterinary Medicine Hannover, 30173 Hannover, Germany; ralph.goethe@tiho-hannover.de; 4 Institute of Clinical Chemistry and Pathobiochemistry, School of Medicine, Technical University of Munich, 81675 Munich, Germany; j.ruland@tum.de; 5 Department of Pathology, University of Veterinary Medicine Hannover, 30559 Hannover, Germany; wolfgang.baumgaertner@tiho-hannover.de

**Keywords:** La Crosse virus, C-type lectin receptor, innate immunity, mincle, CARD9, dendritic cells

## Abstract

La Crosse virus (LACV) is a mosquito-transmitted arbovirus and the main cause of virus-mediated neurological diseases in children. To date, little is known about the role of C-type lectin receptors (CLRs)—an important class of pattern recognition receptors—in LACV recognition. DC-SIGN remains the only well-described CLR that recognizes LACV. In this study, we investigated the role of additional CLR/LACV interactions. To this end, we applied a flow-through chromatography method for the purification of LACV to perform an unbiased high-throughput screening of LACV with a CLR-hFc fusion protein library. Interestingly, the CARD9-associated CLRs Mincle, Dectin-1, and Dectin-2 were identified to strongly interact with LACV. Since CARD9 is a common adaptor protein for signaling via Mincle, Dectin-1, and Dectin-2, we performed LACV infection of Mincle^−/−^ and CARD9^−/−^ DCs. Mincle^−/−^ and CARD9^−/−^ DCs produced less amounts of proinflammatory cytokines, namely IL-6 and TNF-α, albeit no reduction of the LACV titer was observed. Together, novel CLR/LACV interactions were identified; however, the Mincle/CARD9 axis plays a limited role in early antiviral responses against LACV.

## 1. Introduction

La Crosse virus (LACV) is a mosquito-borne member of the California encephalitis group of the order *Bunyavirales* [1,2]. LACV is endemic to Midwestern regions of the USA but its geographical distribution is currently expanding to previously non-affected areas of the USA [3,4]. Children are more susceptible than adults to virus-mediated neurological diseases since the immune system is relatively immature at birth and during childhood [5,6]. LACV is one of the main causative agents of pediatric viral encephalitis in the USA, affecting mostly children under the age of 16 [7,8]. LACV infections account for up to 300,000 infections/year, of which, ~70 cases/year result in a severe outcome like meningitis, encephalitis, or meningoencephalitis [3,7,9].

During natural transmission by the bite of an infected mosquito, LACV encounters dermal innate immune cells, predominantly dendritic cells (DCs), and macrophages at the site of infection [10]. Recognition of LACV by innate immune cells occurs via pattern recognition receptors (PRRs), like retinoic acid-inducible gene I (RIG-I)-like receptor and toll-like receptor 3 (TLR3). RIG-I and TLR3 engagement mediate activation of interferon response factor (IRF)3, IRF7, and nuclear factor-κB (NF-κB), leading to type-I IFN responses and production of proinflammatory cytokines, respectively [11,12]. Thus, these receptors were described to elicit protective immune responses upon LACV infection [12,13,14]. In addition, depletion of myeloid DCs in LACV-infected adult mice resulted in aggravated neurological disease in the central nervous system, thus highlighting the importance of DCs recognition of LACV [14].

C-type lectin receptors (CLRs), an important class of PRRs, are carbohydrate-binding receptors that sense pathogen-associated molecular patterns (nonself-antigens) and danger-associated molecular patterns (self-antigens) [15,16]. Upon antigen recognition, myeloid CLRs expressed by antigen presenting cells (APCs), like DCs and macrophages, trigger a variety of functions that range from uptake, phagocytosis, cytokine production, and antigen presentation to naïve T cells, to shaping of adaptive immune responses [16]. In viral recognition, CLRs have a complex role with an intrinsic duality: they can either elicit antiviral responses by shaping innate and adaptive immune responses or act as entry receptors which enable virus transmission and dissemination [17,18,19]. Recognition of the glycan moieties of viral glycoproteins is decisive for the outcome of virus infections. LACV possesses two viral glycoproteins—Gn and Gc—that mediate attachment to host cells and the initial steps of replication [20,21]. Gn is composed mainly of *N*-glycans of the high-mannose type, while Gc comprises both complex and intermediate types of oligosaccharides [21]. Two entry mechanisms of LACV in mammalian cells were described. LACV can either enter host cells via clathrin-mediated endocytosis allowing Rab-5-mediated trafficking to early endosomes [22] or via the dendritic cell-specific intercellular adhesion molecule 3-grabbing nonintegrin (DC-SIGN) [23], a CLR that is well-described as an entry receptor for different bunyaviruses [24]. However, the role of additional CLRs in the recognition of LACV is still unknown.

To identify CLR candidates in viral recognition, one can evaluate binding to CLRs with expressed and purified viral envelope glycoproteins [25] or by accessing viral infectivity in cells lines overexpressing CLRs [26]. Here, we report an unbiased ELISA-based screening of pure LACV with a CLR-hFc fusion protein library [27] in order to identify novel receptors involved in LACV recognition. Coating of pure virus particles on ELISA plates [28] enabled to detect five novel murine CLRs that recognize LACV: Macrophage-inducible Ca^2+^-dependent lectin receptor (Mincle), Dectin-1, Dectin-2 and, to a lesser extent, macrophage galactose binding lectin (MGL-1) and Langerin. Noteworthy was the binding of LACV to Mincle, Dectin-1, and Dectin-2 which all are CLRs that signal via the caspase recruitment domain family member 9 (CARD9), an adaptor protein that promotes NF-κB activation through multiple innate sensor proteins [29]. Since myeloid dendritic cells play an important role in response to LACV infection [14], we compared the response of Mincle^−/−^ and CARD9^−/−^ bone marrow-derived DCs (BMDCs) after incubation with LACV or Poly(I:C)—a known agonistic TLR3 and RIG-I ligand [30]. Interestingly, the Mincle/CARD9 axis contributed to initial antiviral responses against LACV in BMDCs. However, no differences in virus titers were observed between wild type (WT), Mincle^−/−^, and CARD9^−/−^ DCs at different time points after LACV infection.

## 2. Materials and Methods

### 2.1. Mice

CLR- and CARD9-deficient mice and the respective C57BL/6 wild type (WT) control mice were housed in the animal facility of the University of Veterinary Medicine Hannover under controlled temperature and humidity and specific pathogen-free (SPF) conditions. Food and water were provided ad libitum. Mice were sacrificed and tibia and femur from CLR- and CARD9-deficient or WT mice were prepared to perform extraction of bone marrow cells. Sacrificing of mice for scientific purposes was approved by the Animal Welfare Officers of the University of Veterinary Medicine Hannover (AZ 02.05.2016).

### 2.2. Virus and Cell Culture

Baby hamster kidney cells (BHK-21, clone 13, ATCC^®^ CCL-10) and Vero E6 cells (clone E6, ATCC^®^ CRL-1586) were cultured in IMDM complete medium (PAN-Biotech, Aidenbach, Germany) supplemented with 2 mM l-glutamine, 100 U/mL penicillin, 100 µg/mL streptomycin, and 5% fetal bovine serum (PAN-Biotech). Bone marrow cells were isolated from tibia and femur of C57BL/6 mice as previously described [31]. To obtain BMDCs, bone marrow cells were cultured in IMDM complete medium (with 10% FBS instead) supplemented with 5% of GM-CSF supernatant derived from X63 cells [32]. Medium was exchanged every 48 h and BMDCs were used after 8–10 days of differentiation to ascertain that ≥80% of the cell population expressed the DC differentiation marker CD11c. All cells were grown at 37 °C in 5% CO_2_. La Crosse virus was routinely produced in T-75 or T-125 flasks by infecting Vero E6 cells at a multiplicity of infection (MOI) of 0.01. The infection medium for LACV purification was IMDM complete medium (with 2 mM l-glutamine, 100 U/mL penicillin, 100 µg/mL streptomycin, and 2% FBS). Supernatant containing the virus was collected 2–3 days after infection when ≥80% of cells were lysed. The supernatant was centrifuged at 3000× *g* for 30 min at 4 °C and collected for further purification by flow-through chromatography or stored at −80 °C for further use.

### 2.3. Flow-Through Chromatography

A pericyclic pump (Carl Roth Cyclo II, Karlsruhe, Germany) was used for all liquid chromatography experiments. The liquid chromatography columns used were a 5 mL Capto Q and a 4.7 mL Capto Core 700 (GE Healthcare, Danderyd, Sweden). Capto Q is an anion-exchange chromatography column, while Capto Core 700 is a ligand core chromatography column. The protocol was performed as previously described with some modifications [33,34]. Briefly, a small-scale batch of mock- or LACV-infected Vero E6 cells (100 mL) was sterile-filtered (0.22 µm filter) and concentrated into a final volume of 5 mL using a Spin-X ultrafiltration concentrator of 100 kDa (Corning Inc., Corning, NY, USA). Samples were conditioned with 25 mL of virus dilution buffer (VDB, 20 mM phosphate buffer with 0.5 M NaCl, pH 7.2) prior to loading on the respective liquid chromatography columns. Before sample loading, columns were equilibrated with five column volumes (CV) of VDB. Capto Q and Capto Core 700 were plugged in series and mock- or LACV-infected cell supernatant were loaded on the respective columns at a flow rate of 1 mL/min. Mock- and LACV-infected samples were collected in the flow-through, whereas host cell protein contaminants remained bound to the column. After sample loading, columns were washed with one CV of VDB to elute remaining virus from the column. The Capto Q column was cleaned with 2 M NaCl and 1 M NaOH, while the Capto Core 700 was cleaned with 1 M NaOH and 30% isopropanol (*v*/*v*). After column cleaning, they were equilibrated with 5 CV of VDB to be used in a second round of purification. Purified samples after the first round of purification were loaded again on the respective columns and the flow-through was collected. This second round of purification was to ensure >99% removal of host cell-derived proteins to perform binding studies of highly pure LACV preparations with the CLR-hFc fusion protein library. Finally, samples purified after the two rounds of column chromatography were concentrated to a final volume of 3 mL using a Spin-X UF concentrator (100 kDa MWCO). The virus titer and protein concentrations were determined before and after the purification process. Three independent small-scale batches of mock-infected or LACV-infected Vero E6 cells were prepared and purified by the above described procedure.

### 2.4. CLR-hFc Fusion Protein Production

The expression and purification of the murine and human CLR-hFc fusion protein library was performed as previously reported [27,35]. Briefly, cDNA fragments, amplified by PCR using CLR-specific primers for the CLR extracellular domain, were ligated into a pFuse-hIgG1-Fc expression vector (InvivoGen, Toulouse, France). CHO-S cells were transiently transfected with the CLR-pFUSE-hIgG1-Fc vector and the CLR-hFc fusion protein was purified from the supernatant using a HiTrap protein G column (GE Healthcare). CLR-hFc purity and identity were confirmed by SDS-PAGE followed by Coomassie staining and Western blot using an anti-human IgG-horseradish peroxidase (HRP) antibody (Dianova, Hamburg, Germany). The sequences of the primers used to amplify the cDNA fragments encoding for the extracellular parts of the respective murine and human CLRs are listed in the Supplementary Materials (Table S1).

### 2.5. ELISA-Based LACV/CLR Binding Studies

A half-area microplate (Greiner Bio-one GmbH, Frickenhausen, Germany) was coated overnight at 4 °C with either 50 µL supernatant of purified mock-infected Vero E6 cells or with 50 µL purified supernatant of 1 × 10^7^ FFU/mL LACV-infected Vero E6 cells. Unbound virus was removed by washing three times with washing buffer (PBS 1×, 0.05% Tween-20) and the plate was blocked with 150 µL/well of 1% BSA in PBS (fraction V, IgG free, Thermo Fisher Scientific, Darmstadt, Germany) for 2 h at RT. The plate was again washed three times with washing buffer and 250 ng/well of each CLR-hFc fusion protein in lectin binding buffer (50 mM HEPES, 5 mM MgCl_2_, 5 mM CaCl_2_) was added. After 1h incubation at RT, the plate was washed and incubated with an anti-human IgG-horseradish peroxidase (HRP) antibody (Dianova, Hamburg, Germany) at a 1:5000 dilution in diluent buffer (0.1% BSA in PBS, 0.05% Tween-20) for 1 h at RT. After a final washing step, 50 µL/well of the substrate solution was added (*O*-phenylenediamine dihydrochloride substrate tablet (Thermo Fisher Scientific, Dreieich, Germany), 24 mM citrate buffer, 50 mM phosphate buffer, and 0.04% H_2_O_2_ in H_2_O) and color development was stopped with 2 M sulfuric acid. Absorbance at 495 nm was measured using a Multiskan GO microplate spectrophotometer (Thermo Fisher Scientific). Four independent binding experiments with the CLR-hFc fusion protein library were performed (triplicates each).

### 2.6. Real-Time PCR

BMDCs from Mincle^−/−^, CARD9^−/−^ and WT mice were seeded in a 6-well plate at 1 × 10^6^ cells/well and infected with LACV at a MOI of 5 or 20. Upon addition of the virus, supernatants were collected to assess virus titers at the time point 0 h. After 2 h of incubation at 37 °C and 5% CO_2_, cells were centrifuged at 300× *g* for 5 min at 4 °C, and supernatant was collected for virus titer measurement. The cell pellet was washed three times with PBS to remove unbound virus and 750 µL of Qiazol was added (baseline for internalized LACV). In samples taken at later time points of LACV infection, namely 8 h and 24 h, the same washing procedure was performed at time point 2 h to remove unbound virus. After 8 h and 24 h of incubation, cells were washed three times and suspended in Qiazol. Total RNA was isolated from infected DCs with the RNeasy extraction kit (Qiagen, Hilden, Germany) according to manufacturer’s instructions. For RT-PCR, the One-Step RT-PCR kit (Qiagen) was used with an amount of RNA template of 60 ng per sample. Expression levels were measured using an AriaMx Real-Time PCR system (Agilent Technologies, Santa Cruz, CA, USA). Levels of LACV nucleoprotein (N) RNAs were normalized to the expression of the housekeeping 18S RNA. To evaluate differences in LACV nucleoprotein levels in Mincle^−/−^ and CARD9^−/−^ DCs against WT DCs, the δCt values of Mincle^−/−^ and CARD9^−/−^ DCs were subtracted from the δCt value of the WT DCs at their respective time points. Expression fold change was then calculated by the δδCT method. In WT DCs, expression of Mincle and CARD9 upon LACV infection was evaluated at different time points, using the housekeeping 18S RNA for normalization. The primers and probes used for LACV [36] were forward primer (FW) 5′-GTGTTTTATGATGTCGCATCA-3′, reverse primer (RV) 5′-CATATACCCTGCATCAGGATCAA-3′, fluorescently-labeled probe 6-carboxyfluorescein (Fam)-CAGGTGCAAATGGA-minor groove binder moiety (MGB). The murine 18S, Mincle, and CARD9 primers and probe mix were acquired from Thermo Fisher Scientific (Mm03928990_g1, FAM-MGB; Mm01183703 m1, FAM-MGB; Mm01327594 m1, FAM-MGB). For all RT-PCR experiments, three independent infection experiments were performed (duplicates each).

### 2.7. LACV Infection of BMDCs

BMDCs from Mincle^−/−^, CARD9^−/−^, and WT mice were seeded in a 96-well plate at 8 × 10^4^ cells/well in IMDM complete medium on the day prior to infection. One hour prior to infection, cells were either pretreated with 25 µg/mL of high molecular weight (HMW) Poly (I:C) (InvivoGen) or left mock-treated [37]. After 1 h, 100 µL of LACV at MOI 5 or 20 suspended in IMDM complete medium were added to the pretreated or mock-treated cells. After 8 h and 24 h of infection, plates were centrifuged at 300× *g* for 5 min at 4 °C, and supernatants were collected for measurement of the LACV titer and cytokine production. The cell pellet was washed two times with FACS buffer (PBS, 2% FBS, 1 mM EDTA) and suspended in 50 µL of anti-mouse CD16/32 (Fc-blocking antibody, dilution 1:100, clone 93, Thermo Fisher Scientific) for 15 min at 4 °C. Afterwards, cells were incubated in FACS buffer containing APC-conjugated anti-CD11c antibody (1:250 dilution, clone N418, Thermo Fisher Scientific) in combination with either one of the following antibodies for 25 min at 4 °C in the dark: FITC-conjugated anti-major histocompatibility complex (MHC)-I H-2Kb antibody (1:100 dilution, clone AF6-88.5.5.3, Thermo Fisher Scientific), FITC-conjugated anti-MHC-II I-antibody (1:100 dilution, clone AF6-120.1, Thermo Fisher Scientific), or FITC-conjugated anti-CD80 antibody (1:100 dilution, clone 16-10A1, Thermo Fisher Scientific). The cell pellet was suspended in 2% PFA for 1 h at RT for virus inactivation. Flow cytometry measurements were performed using the Attune NxT flow cytometer (Thermo Fisher Scientific). In all flow cytometry assays, cells were hierarchically gated to exclude debris, followed by single cell gating, gating on CD11c^+^ cells, and gating on cells expressing either MHC-I, MHC-II, or CD80. Flow cytometry data were analyzed using the FlowJo version 10 software (Tree Star Inc., Ashland, OR, USA). Three independent flow cytometry experiments were performed (duplicates each).

### 2.8. Cytokine ELISAs

DC supernatants collected after LACV stimulation were analyzed for the proinflammatory cytokines IL-6 and TNF-α according to the manufacturer’s instructions (DuoSet ELISA kits, R&D Systems, Minneapolis, MN, USA). Plates were developed with the substrate 3,3′,5,5′-tetramethylbenzidine (TMB) and the color reaction was stopped with 2 M sulfuric acid. Absorbance was measured at 450 nm with a wavelength correction at 570 nm using a Multiskan Go microplate spectrophotometer. Murine interferon-β (IFN-β) luminescence ELISA was performed according to the manufacturer’s instructions (Lumikine™ Xpress mIFN-β, InvivoGen). Plates were read using a Tecan^®^ Infinite 200 Pro. Three independent experiments were performed (triplicates each). 

### 2.9. In Vitro Viral Replication

Samples containing LACV were evaluated for viral titers by performing endpoint dilution assay in BHK-21 cells. Briefly, a single 96-well plate was used per LACV supernatant sample and all wells of the plate were filled with IMDM medium (supplemented with 2 mM l-glutamine, 100 U/mL penicillin, 100 µg/mL streptomycin, and 5% FBS) prior to addition of the virus sample. Then LACV supernatant was diluted 1:10 and added in the first column of the 96-well plate (8 technical replicates). Virus was then serially diluted along the 96-well plate, except for the last two columns (negative control). Next, 100 µL/well of a BHK-21 cells suspension was added to all wells and cells were incubated 4–6 days at 37 °C in 5% CO_2_. The 50% tissue culture infective dose (TCID_50_) was obtained and focus forming units (FFU/mL) were estimated using the formula 0.69 × TCID_50_, as described previously [38]. 

### 2.10. Transmission Electron Microscopy

WT BMDCs were seeded in a 6-well plate at 1 × 10^6^ cells/well and infected with LACV at MOI 20. After 2 h of incubation at 37 °C and 5% CO_2_, cells were washed three times with 1× PBS, and the cell pellet was fixed overnight in 2.5% glutaraldehyde at 4 °C. After fixation, samples were processed as previously described [39,40]. Finally, ultrathin sections were cut with a diamond knife (Diatome, Hatfield, UK) and transferred to copper grids. For ultrastructural analysis, a transmission electron microscope (EM 10C, Zeiss, Munich, Germany) was used.

### 2.11. Protein Concentration Determination

Protein concentration was calculated using the Pierce Protein Assay kit (Thermo Fisher Scientific) according to the manufacturer’s instructions. Each sample was tested in duplicate and absorbance was measured at 562 nm using a Multiskan Go microplate spectrophotometer.

### 2.12. Statistical Analysis

Statistical analysis was performed using the GraphPad Prism 7 software (GraphPad, San Diego, CA, USA). Data are represented as a mean ± SEM for all experiments. A two-way ANOVA with a Tukey’s honest significance test was used in the data treatment (*p* < 0.05 was considered statistically significant).

## 3. Results

### 3.1. LACV Purification by Flow-through Chromatography

**Figure 1:**
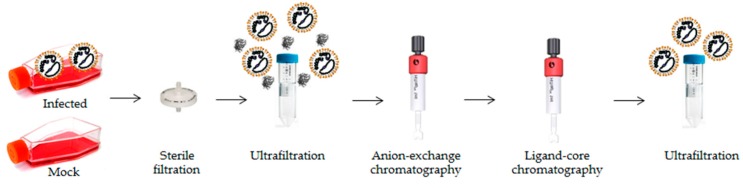
Schematic representation of the flow-through chromatography process used for La Crosse virus (LACV) purification. Mock- and LACV-infected supernatants of VeroE6 cells were purified by sequential steps of ultrafiltration and liquid chromatographic processes to remove host-cell derived proteins (HCPs). At the end of the purification process, a virus enrichment in the LACV sample is attained by efficient removal of HCPs.

To evaluate LACV/CLR interactions, highly pure virus samples were required, since CLRs are known to interact with glycoproteins or other glycoconjugates from damaged and necrotic cells [16]. Therefore, to obtain highly pure LACV particles, a flow-through chromatography method was established (Figure 1). In this method, host cell-derived proteins (HCPs) and DNA bind to the stationary phase of the column (resin), while the virus particles are obtained in the flow-through of the column [33,34].

By purifying supernatants of LACV-infected or mock-infected Vero E6 cells through a two-step sequential flow-through chromatography process, 99.4% of HCPs were efficiently removed (Table 1). Moreover, an endpoint dilution assay of pure LACV samples confirmed that the virus remained infectious as a final virus titer of 4.3 ± 1.5 × 10^7^ FFU/mL was determined (Table 1). Thus, although the remaining viral infectivity was only 1.9% of the original infectivity, a viral enrichment of at least 3-fold was successfully achieved at the end of the purification process.

### 3.2. ELISA-Based Screening of LACV/CLR Interactions

**Figure 2:**
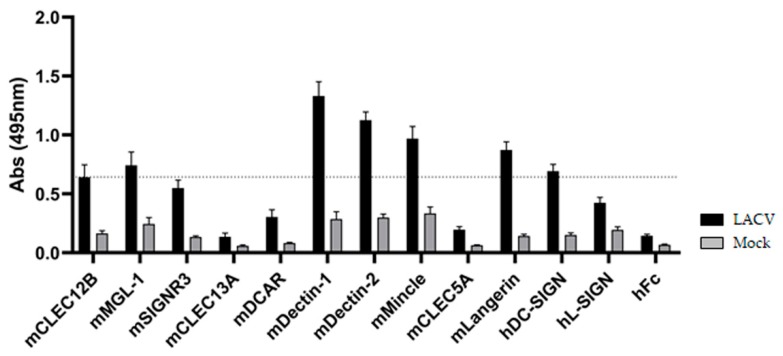
ELISA-based screening of LACV with a C-type lectin receptor (CLR)-hFc fusion protein library. DC-SIGN was reported to recognize Gc/Gn of LACV [23] and is considered a positive control. To discard possible false-positives, a 3-fold margin in the absorbance value relative to hFc (negative control) was given (dotted line), based on previous screenings with the CLR-hFc library [27]. Data depicted are the mean ± SEM of four independent experiments (duplicates each).

To identify novel LACV/CLR interactions (Figure 2), purified LACV was coated on ELISA plates and probed with a comprehensive CLR-hFc fusion protein library [27,35]. This library was extended with the addition of novel members to perform CLR/viral screening, such as CLEC5A—a CLR involved in exacerbated proinflammatory responses in dengue virus infection [41]; Langerin—a CLR responsible for antiviral responses against HIV-1 in DCs [42], and L-SIGN (also known as DC-SIGNR), which is described to function as an attachment factor for other bunyaviruses, such as the Rift Valley fever virus [43]. The purified mock control was also coated on the plate and served to account for unspecific binding of the CLR-hFc fusion proteins to remaining HCPs. 

Since DC-SIGN was previously described to bind to the Gc/Gn glycoproteins of LACV and to act as an entry receptor for the virus [23], DC-SIGN was included as a positive control. As shown in Figure 2, besides DC-SIGN-hFc, the ELISA-based screening identified five novel candidate CLRs that bind to LACV: Dectin-1, Dectin-2, Mincle and, to a lesser extent, MGL-1 and Langerin.

### 3.3. LACV Is Internalized by DCs

Dectin-1, Dectin-2, and Mincle are CLRs that share canonical signaling via spleen tyrosine kinase (Syk) and the adaptor protein CARD9 that is part of the CARD-BCL10-MALT1 complex [29,44]. Assembling of this ternary complex ultimately leads to NF-κB activation. Since the role of Mincle- and CARD9-mediated innate responses in LACV infection has not yet been described to the best of our knowledge, we further examined these findings by performing in vitro DC stimulation assays using Mincle^−/−^, CARD9^−/−^, and WT DCs.

**Figure 3:**
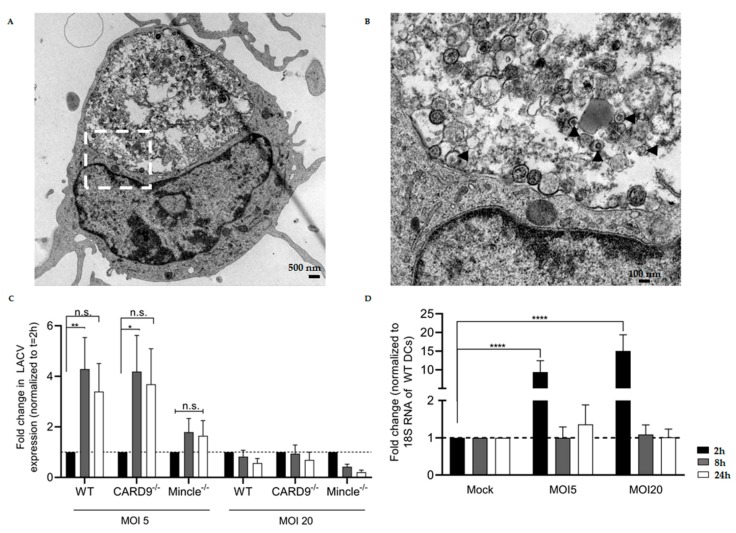
LACV internalization, replication in Mincle^−/−^, CARD9^−/−^, or WT dendritic cells (DCs), and LACV-dependent induction of Mincle expression. (**A**) Transmission electron microscopy (TEM) picture showing a LACV-infected WT DC at time 2 h (magnification 6300×). (**B**) Close-up TEM picture (magnification 25,000×) of highlighted region in (A). The black arrowheads show LACV particles inside vesicles in the phagolysosome. (**C**) Expression levels of LACV N mRNA at different time points in DCs. The time point 2 h was used as baseline (internalized LACV). In C, two distinct LACV MOI were used—MOI 5 and MOI 20. (**D**) Expression levels of Mincle mRNA at different time points in LACV-infected WT DCs. The mock-infected DCs were used as baseline. Data shown in (C) and (D) are mean ± SEM and three independent experiments were performed. A two-way ANOVA with a Tukey’s honest significance test was used to compare differences between the different groups and *p* < 0.05 was considered significant (* *p* < 0.05, ** *p* < 0.01, *** *p* < 0.001, **** *p* < 0.0001).

In order to assess the role of Mincle and CARD9 in LACV infection, first we evaluated if LACV was internalized into WT DCs. Transmission electron microscopy (TEM) analysis after short-term LACV infection (MOI 20) allowed us to observe that LACV particles are surrounded by small vesicles to larger endosomal structures reminiscent of phagolysosomes inside WT DCs, thus suggesting elimination of virus particles (Figure 3A,B). Since LACV is internalized by WT DCs, we then investigated if LACV was able to replicate in Mincle^−/−^, CARD9^−/−^, and WT DCs by RT-PCR. LACV was added at two different MOI (5 and 20) for 2 h to allow for virus attachment and internalization into the cells. After 2 h, washing of unbound viruses was performed, followed by incubation until time point 8 h or 24 h.

RT-PCR results showed replication of LACV in WT and CARD9^−/−^ DCs at MOI 5 (Figure 3C), indicating that the virus seems capable of initializing replication inside DCs (*p* < 0.05 by a two-way ANOVA test). However, after 24 h, no increase in the mRNA levels of LACV nucleoprotein occurred, suggesting that LACV replication is blocked in DCs. Surprisingly, at MOI 20, no LACV replication inside DCs seemed to take place at all. It should be noted that results were confirmed in three replicate experiments. The fact that no LACV replication at MOI 20 was observed may suggest saturation of infection possibly due to a more prominent DC activation, thus preventing the initialization of replication.

In WT DCs, LACV-dependent upregulation of Mincle expression was observed (*p* < 0.05 by a two-way ANOVA test) after 2 h of infection (Figure 3D). This finding supports the identified interaction between Mincle-hFc and LACV by ELISA, as it demonstrates induction of Mincle expression at an early time point of infection. It is noteworthy that the expression level of the adaptor protein CARD9 remained unaltered upon LACV infection of WT DCs (Figure S1).

### 3.4. Activation of DCs by LACV

**Figure 4:**
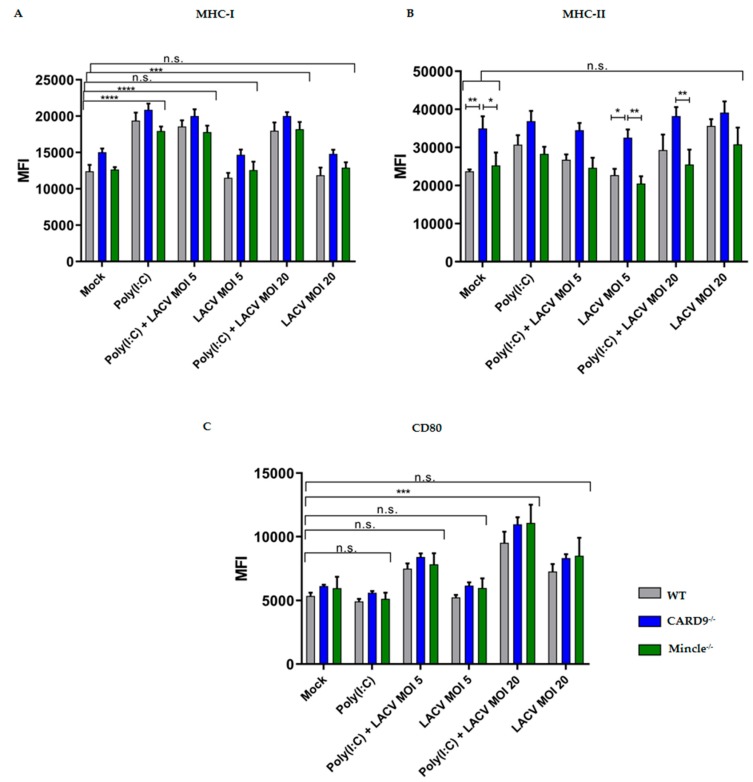
Effector functions of Mincle^−/−^, CARD9^−/−^, and WT DCs stimulated with LACV. (**A**) Surface expression of MHC-I. (**B**) Surface expression of MHC-II. (**C**) Surface expression of CD80 in Mincle^−/−^, CARD9^−/−^ or WT DCs after 24 h of stimulation. Data represented are mean ± SEM of three independent experiments. Data are presented as MFI (median fluorescence intensity) values. A two-way ANOVA with a Tukey’s honest significance test was performed and *p* < 0.05 was considered significant (* *p* < 0.05, ** *p* < 0.01, *** *p* < 0.001, **** *p* < 0.0001).

To better understand whether the absence of Mincle and CARD9 affected DC maturation, we stimulated DCs with LACV (MOI 5 or 20) or with mock-infected supernatant from Vero E6 cells (negative control). Since glycan-based signaling via CLRs often requires coactivation with other PRRs, we included Poly(I:C) to prime activation [45,46]. Hence, we pretreated Mincle^−/−^, CARD9^−/−^, and WT DCs with Poly(I:C) for 1 h [37] before adding LACV at MOI 5 or 20 (Figure 4). After 24 h of LACV stimulation, MHC-I expression was significantly higher (*p* < 0.001 by a two-way ANOVA test) in DCs costimulated with Poly(I:C) and LACV (MOI 5 or 20) compared to the mock control, while stimulation with LACV only at both MOI induced no significant differences (Figure 4A).

While the upregulated MHC-I expression was due to Poly(I:C) stimulation only, surface expression of the costimulatory molecule CD80 was significantly increased (*p* < 0.001 by a two-way ANOVA test) in DCs costimulated with Poly(I:C) and LACV MOI 20, when compared with Poly(I:C) alone or mock control (Figure 4C). This finding indicates a synergistic mode of action of Poly(I:C) and LACV on the expression of this costimulatory molecule. The expression of MHC-II in CARD9^−/−^ DCs was significantly higher (*p* < 0.001 by a two-way ANOVA test) in three different conditions (mock, LACV MOI 5, costimulation with Poly(I:C) and LACV MOI 20) in comparison to WT and/or Mincle^−/−^ DCs (Figure 4B). No differences in MHC-II expression were observed between mock treated and/or LACV infected WT, CARD9^−/−^, and Mincle^−/−^ DCs, indicating that neither LACV nor costimulation with Poly(I:C) and LACV affected MHC-II expression (Figure 4B). It is noteworthy that Mincle^−/−^ and CARD9^−/−^ DCs did not exhibit a different expression of MHC-I and CD80 in all conditions tested, when compared to WT DCs (*p* > 0.05 a by two-way ANOVA test). DC effector functions were also assessed at an earlier time point of infection (after 8 h), at which CARD9^−/−^ and WT DCs exhibited upregulated MHC-I and MHC-II expression in DCs costimulated with Poly(I:C) and LACV (Figure S2). No differences were observed in CD80 expression in Mincle^−/−^, CARD9^−/−^, and WT DCs after 8 h of infection (Figure S2). Since an upregulated CD80 expression upon stimulation with Poly(I:C) and LACV (MOI 20) was observed in WT, Mincle^−/−^ and CARD9^−/−^ DCs after 24 h (Figure 4), Mincle/CARD9 mediated signaling seems to be dispensable for the expression of costimulatory molecules by DCs. 

### 3.5. Mincle and CARD9 Contribute to Proinflammatory Signaling of LACV Infected DCs

**Figure 5:**
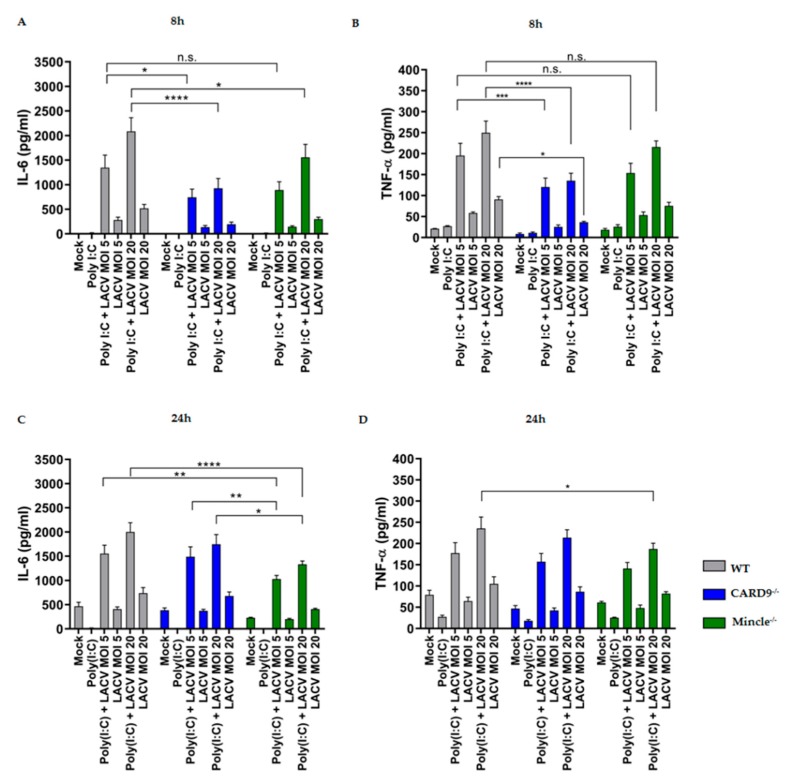
Mincle^−/−^ and CARD9^−/−^ DCs display impaired proinflammatory cytokine production upon LACV challenge in the presence of Poly(I:C). IL-6 and TNF-α production after 8 h (**A**,**B**, respectively) and 24 h of stimulation (**C**,**D**, respectively). Data represented are mean ± SEM of three independent experiments. A two-way ANOVA with a Tukey’s honest significance test was performed and *p* < 0.05 was considered significant (* *p* < 0.05, ** *p* < 0.01, *** *p* < 0.001, **** *p* < 0.0001).

The above data show the limited capacity of LACV to prime DCs maturation. Since the proinflammatory response of DCs might contribute to antiviral immunity against LACV, we performed cytokine ELISA for hallmark cytokines of proinflammatory responses, namely, TNF-α and IL-6 after 8h and 24h of virus infection (Figure 5). To boost cytokine expression, we also incubated cells with Poly(I:C) and LACV. As shown in Figure 5, a substantial production of IL-6 and, to a lesser extent, of TNF-α were observed in DCs costimulated with Poly(I:C) and LACV (MOI 5 and 20) for 8 h and 24 h. Mincle^−/−^ and CARD9^−/−^ DCs exhibited an impaired proinflammatory cytokine production at an earlier time point (*t* = 8 h), as indicated by a decreased IL-6 production (*p* < 0.05 by a two-way ANOVA test), when compared to WT DCs (Figure 5A,B). Interestingly, only Mincle^−/−^ DCs presented a prominent reduction in IL-6 production after 24 h (Figure 5C) of costimulation with Poly(I:C) and LACV (MOI 5 and 20) compared to WT DCs (*p* < 0.01 by a two-way ANOVA test). Moreover, in Mincle^−/−^ DCs decreased TNF-α production was observed after LACV challenge for 24 h, while in CARD9^−/−^ DCs this effect was only noticed at an earlier time point (Figure 5B,D). In both, differences were only detected in the Poly(I:C) and LACV MOI 20 challenge (*p* < 0.05 by a two-way ANOVA test). It is noteworthy that the impaired proinflammatory response was dependent on the presence of both LACV and Poly(I:C), since Poly(I:C) alone resulted in a low IL-6 and TNF-α production. This observation suggests a synergistic mode of action of Poly(I:C) and LACV on the production of proinflammatory cytokines by DCs in a partially Mincle/CARD9-dependent manner.

Type-I interferon responses in mice were shown to be crucial for LACV clearance and reduction of LACV-induced neurological diseases [14,47,48]. In the LACV stimulation experiments using Mincle^−/−^, CARD9^−/−^, and WT DCs, a decreased type-I interferon-β (IFN-β) production could be detected only in CARD9^−/−^ DCs by luminescence IFN-β ELISA (Figure S3).

**Figure 6:**
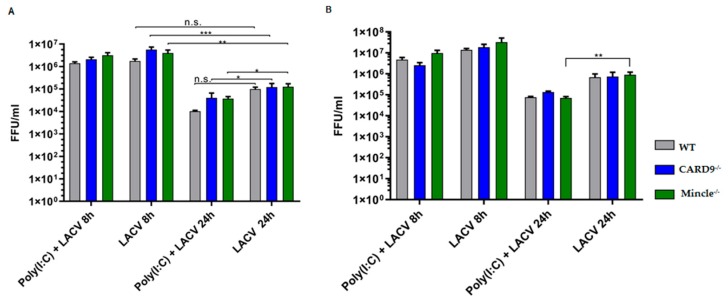
Viral loads in Mincle^−/−^, CARD9^−/−^, and WT DCs challenged with LACV. Virus titer in the supernatant of Mincle^−/−^, CARD9^−/−^ and WT DCs infected with LACV at a MOI 5 (**A**) and a MOI 20 (**B**) for 8 h and 24 h. Data shown are mean ± SEM of three independent experiments. A two-way ANOVA with a Tukey’s honest significance test was employed and *p* < 0.05 was considered significant (* *p* < 0.05, ** *p* < 0.01, *** *p* < 0.001).

To evaluate if the impaired proinflammatory and IFN-β response was associated with a decreased antiviral response by Mincle^−/−^ and CARD9^−/−^ DCs, we analyzed the viral titer after 8 h and 24 h of incubation with LACV (Figure 6). The results showed a considerable reduction (>1 log) of the LACV titer at MOI 5 from 8 h to 24 h (Figure 6A), indicating that LACV does not release infectious progeny within 24 h of DCs challenge (*p* < 0.01 by a two-way ANOVA test). Therefore, albeit the increase in LACV N mRNA levels in WT and CARD9^−/−^ DCs (Figure 3C), no productive DCs infection was observed.

When Poly(I:C) was added, a lower LACV titer (*p* < 0.05 by two-way ANOVA test) was attained in Mincle^−/−^ DCs at MOI 5 or 20 (Figure 6A,B). Such a reduction was also observed in CARD9^−/−^ DCs at a MOI 5 (Figure 5A). Indeed, Poly(I:C) activates TLR3 and RIG-I pathways [45] and is well-documented to enhance antiviral responses of host immune cells [49,50,51]. Poly(I:C)-dependent LACV titer reduction in primary glial cells was reported already in 1975, though the mechanism was still unknown at that time [52]. Finally, no differences in LACV titers were observed between WT and Mincle^−/−^ or CARD9^−/−^ DCs, indicating that the Mincle/CARD9 axis plays a limited role in DCs infectivity.

## 4. Discussion

So far, the role of PRRs in the recognition of LACV has been elusive. To this end, in this study we have addressed the recognition of LACV by host CLRs, an important class of PRRs that initiate innate immune responses. La Crosse virus is the leading causative agent of neuroinvasive viral disease in younger children, accounting for up to 55% of all reported cases [7]. In the upcoming years, expansion of LACV vector mosquitoes can result in an increased risk of exposure for the high-risk groups within the population [9,53,54].

CLR/viral interactions can either trigger antiviral responses resulting in an efficient viral clearance or viruses can subvert host defense by covering their surface with highly glycosylated proteins to promote attachment and internalization into host cells, followed by replication and dissemination [17,18]. The only described interaction of LACV with CLRs is the DC-SIGN recognition of pseudotypes bearing the Gn/Gc glycoproteins of LACV, which resulted in increased viral entry in Raji B cells expressing DC-SIGN [23].

To identify novel CLR/viral interactions, we established, in the first place, a flow-through chromatography process for LACV purification, which allowed us to obtain highly pure virus samples with minimal HCP contaminants. Flow-through chromatography purification of influenza viruses or reoviruses was previously shown to be a powerful tool to remove protein and DNA impurities to more than 90% and 95%, respectively [33,34]. In our approach, we performed an additional round of purification through the anion-exchange (Capto Q) and ligand-core (Capto Core 700) chromatography columns to ensure that only marginal amounts of HCPs would be present in the purified samples. This resulted in a decrease in the final LACV virus titer, but it was a necessary trade-off to reach over 99% removal of HCPs with a virus enrichment of at least 3-fold. The trace amounts of HCPs obtained is a crucial step to perform ELISA-based screening with our CLR-hFc fusion protein library [27,35], to avoid the presence of glycoproteins or glycan structures associated with damaged and necrotic cells [55]. CLR recognition of endogenous ligands released from dead and/or dying cells include sensing of F-actin by DNGR-1 [56], binding of CLEC12A to uric acid crystals [57], uptake of apoptotic cells by MGL-1 [58], and recognition by Mincle of SAP130 [59], cholesterol crystals [60] and β-glucosylceramide [61]. Therefore, virus enrichment and maximum removal of HCPs were crucial to avoid interactions that could mask binding events between LACV and the CLR-hFc fusion proteins. 

By coating highly pure LACV- or mock-infected supernatant (control) from Vero E6 cells in a plate and performing an ELISA-based high-throughput screening with the CLR-hFc fusion protein library, we discovered five novel LACV-CLRs interactions: Dectin-1-hFc, Dectin-2-hFc, Mincle-hFc, and, to a lesser extent, Langerin-hFc and MGL-1-hFc. Langerin is a CLR that possesses a dual role in viral recognition since it was described to be involved in capturing and eliminating HIV-1 in Langerhans cells [42] or to function as an entry receptor for binding and internalization of influenza A virus (IAV), ultimately leading to increased viral spread [62]. MGL-1 is associated with viral attachment and entry, namely of Ebola virus [63] and IAV [64]. The identification of CARD9-associated CLRs (Dectin-1, Dectin-2, and Mincle) prompted us to stimulate CARD9^−/−^ and Mincle^−/−^ DCs with LACV in vitro. Mincle is a receptor that recognizes pathogen-associated molecular patterns from bacteria [65] and fungi [66] and damage-associated molecular patterns from damaged cells [67]. Dectin-1 and Dectin-2 are mainly associated with a protective effect against fungi [68,69,70], though microbial or endogenous ligands have also been described [29]. The roles of Mincle and Dectin-1 or Dectin-2 in recognition of viruses remain elusive. Since Dectin-1 and Dectin-2 are known mainly as fungal receptors [70], we focused on Mincle in this study. Interestingly, the upregulated Mincle expression at an early time point of LACV infection (2 h) may support the involvement of this CLR in LACV recognition. Nonetheless, by including CARD9^−/−^ DCs we also covered the CARD9-associated CLRs, Dectin-1, and Dectin-2, since they require CARD9 signaling for initiating innate immune responses (see also a scheme showing the signal pathway induced by engagement of Mincle, Dectin-1, and Dectin-2 in Figure S4). 

To confirm LACV internalization, TEM was employed after short-term LACV infection of WT DCs. TEM analysis revealed internalized LACV particles in small to larger endosomal structures, as described for other bunyaviruses, such as the Uukuniemi virus (UUKV) [24]. LACV presence in vesicles reminiscent of phagolysosomes may suggest clearance of virus particles by WT DCs, which is in accordance with the results obtained for the decreased virus titers in WT DCs within 24 h.

DCs stimulation with LACV and Poly(I:C) elicited a similar activation of CD80 expression across Mincle^−/−^, CARD9^−/−^, and WT DCs at MOI 20, though only Mincle^−/−^ DCs presented a decreased proinflammatory cytokine production (IL-6 and TNF-α) upon stimulation with Poly(I:C) and LACV after 24h. CARD9^−/−^ DCs only exhibited impaired IL-6 and TNF-α production under the same conditions at an earlier time point (*t* = 8h). Mincle deficiency is reported to cause significantly impaired proinflammatory responses in vitro and in vivo against the fungi Malassezia [71], group A Streptococcus [65], and *Mycobacterium tuberculosis* [72]. Regarding CARD9, this adaptor protein was shown as an essential component for the RIG-I-dependent proinflammatory response against RNA viruses [73]. A CARD9-dependent impaired IFN-β production was reported by del Fresno et al. [74], where CARD9^−/−^ DCs stimulated with heat-killed *Candida albicans* showed lower IFN-β production in vitro. In the same study, Mincle^−/−^ DCs did not exhibit a lower IFN-β production upon *C. albicans* stimulation in vitro when compared to WT DCs. Our LACV infection results are in accordance with the reported impaired IFN-β production in CARD9^−/−^ DCs. Moreover, we also observed no significant differences in IFN-β responses between Mincle^−/−^ and WT DCs, upon LACV challenge. However, the impaired proinflammatory response and/or IFN-β production displayed by Mincle^−/−^ and CARD9^−/−^ DCs did not affect LACV titers in the supernatant. In WT and CARD9^−/−^ DCs, LACV replication is initiated (at MOI 5), as exemplified by the increase in LACV N mRNA. Surprisingly, no LACV replication was observed at MOI 20, which may have been caused by a more prominent DC activation due to the higher MOI, thus preventing initialization of replication. Similar effects have been described for the *Aureococcus anophagefferens*-Brown Tide virus (AaV) where a saturation of infection was observed at a MOI of 8 [75]. Furthermore, a recent study modeling the replication of Influenza A virus in cell culture demonstrated that at high MOIs the number of defective interfering particles (DIPs) present in the inoculum plays a greater role [76]. DIPs have been shown to compete with intact virus particles for cellular resources, and therefore may significantly reduce viral replication [77]. However, a titration over a broader range would be needed to confirm this hypothesis. As observed for Mincle^−/−^ and CARD9^−/−^ DCs, WT DCs virus titer analysis also confirmed that LACV did not release infectious viral particles into the supernatant within 24 h. DCs were described to support infection of other bunyaviruses, such as Rift Valley fever virus and UUKV, though the production of infectious particles in UUKV was minimal [24]. Thus, our study shows that murine DCs cannot be productively infected with LACV.

Taken together, though our results do not formally exclude that the Mincle/CARD9 axis in myeloid DCs may play a role in early innate responses against LACV, this role seems to be rather limited since no differences in viral elimination between Mincle^−/−^, CARD9^−/−^, and WT DCs were observed. In a previous study on the role of CARD9 in proinflammatory cytokine production during viral infections [78], in vitro stimulation of CARD9^−/−^ DCs with influenza A virus (IAV) also showed an impaired IL-6 and TNF-α production. However, CARD9-mediated innate responses in pulmonary DCs were dispensable for protection against IAV in a murine infection model [78]. 

In conclusion, our results provide insights into LACV recognition by CLRs by establishing a novel high-throughput ELISA-based screening of LACV/CLR interactions. We were able to identify novel CLR/LACV interactions with Mincle, Dectin-1, and Dectin-2 as prominent candidate receptors. Mincle^−/−^ and CARD9^−/−^ DCs exhibited impaired proinflammatory cytokine production, namely IL-6 and TNF-α, upon LACV stimulation. Since the LACV titer in the supernatant of infected Mincle^−/−^ and CARD9^−/−^ DCs was similar to WT DCs, the Mincle/CARD9 axis may play a limited role in antiviral responses against LACV. The functional role of additional CLRs identified by our ELISA as interaction partners of LACV should be addressed in future studies. 

## Figures and Tables

**Table 1:** Evaluation of LACV purification by flow-through chromatography. Supernatants of LACV-infected or mock-infected Vero E6 cells were purified by flow-through chromatography and protein concentrations and virus titers were determined at the beginning and the end of the purification process. The results are the mean ± S.D. of three independent batches.

Sample	Volume (mL)	Protein (µg/mL)	Total Protein (µg)	FFU/mL	Protein Removal (%)
LACV (initial)	100	2600 ± 1200	260,000	2.3 ± 1.8 × 10^9^	-
**LACV (final)**	3	17 ± 5.5	51	4.3 ± 1.5 × 10^7^	99.4
Mock (initial)	100	2450 ± 1100	245,000	0	-
**Mock (final)**	3	8.5 ± 0.2	25.5	0	99.7

## Supplementary Materials

The following are available online at https://www.mdpi.com/1999-4915/11/3/303/s1, Table S1: Table of primers used to amplify the extracellular domain of the mouse and human C-type lectins. Figure S1: CARD9 mRNA expression levels in LACV-infected WT DCs. Figure S2: Effector functions of Mincle^−/−^, CARD9^−/−^, and WT DCs after 8h of LACV infection. Figure S3: CARD9^−/−^ DCs exhibit a decreased IFN-β production. Figure S4: Engagement of CARD9-associated CLRs and downstream signaling cascades.

## References

[1] McJunkin J.E., de los Reyes E.C., Irazuzta J.E., Caceres M.J., Khan R.R., Minnich L.L., Fu K.D., Lovett G.D., Tsai T., Thompson A. (2001). La Crosse Encephalitis in Children. N. Engl. J. Med..

[2] Adams M.J., Lefkowitz E.J., King A.M.Q., Harrach B., Harrison R.L., Knowles N.J., Kropinski A.M., Krupovic M., Kuhn J.H., Mushegian A.R. (2017). Changes to taxonomy and the International Code of Virus Classification and Nomenclature ratified by the International Committee on Taxonomy of Viruses (2017). Arch. Virol..

[3] Haddow A.D., Odoi A. (2009). The Incidence Risk, Clustering, and Clinical Presentation of La Crosse Virus Infections in the Eastern United States, 2003–2007. PLoS ONE.

[4] Miller A., Carchman R., Long R., Denslow S.A. (2012). La Crosse Viral Infection in Hospitalized Pediatric Patients in Western North Carolina. Hosp. Pediatr..

[5] Prendergast A.J., Klenerman P., Goulder P.J.R. (2012). The impact of differential antiviral immunity in children and adults. Nat. Rev. Immunol..

[6] Simon A.K., Hollander G.A., McMichael A. (2015). Evolution of the immune system in humans from infancy to old age. Proc. Biol. Sci..

[7] Gaensbauer J.T., Lindsey N.P., Messacar K., Staples J.E., Fischer M. (2014). Neuroinvasive Arboviral Disease in the United States: 2003 to 2012. Pediatrics.

[8] Haddow A.D., Bixler D., Odoi A. (2011). The spatial epidemiology and clinical features of reported cases of La Crosse Virus infection in West Virginia from 2003 to 2007. BMC Infect. Dis..

[9] Haddow A.D., Jones C.J., Odoi A. (2009). Assessing Risk in Focal Arboviral Infections: Are We Missing the Big or Little Picture?. PLoS ONE.

[10] Albornoz A., Hoffmann A.B., Lozach P.-Y., Tischler N.D. (2016). Early Bunyavirus-Host Cell Interactions. Viruses.

[11] Verbruggen P., Ruf M., Blakqori G., Överby A.K., Heidemann M., Eick D., Weber F. (2011). Interferon antagonist NSs of La Crosse virus triggers a DNA damage response-like degradation of transcribing RNA polymerase II. J. Biol. Chem..

[12] Mukherjee P., Woods T.A., Moore R.A., Peterson K.E. (2013). Activation of the innate signaling molecule MAVS by bunyavirus infection upregulates the adaptor protein SARM1, leading to neuronal death. Immunity.

[13] Weber M., Gawanbacht A., Habjan M., Rang A., Borner C., Schmidt A.M., Veitinger S., Jacob R., Devignot S., Kochs G. (2013). Incoming RNA virus nucleocapsids containing a 5’-triphosphorylated genome activate RIG-I and antiviral signaling. Cell Host Microbe.

[14] Taylor K.G., Woods T.A., Winkler C.W., Carmody A.B., Peterson K.E. (2014). Age-dependent myeloid dendritic cell responses mediate resistance to la crosse virus-induced neurological disease. J. Virol..

[15] Mayer S., Raulf M.-K., Lepenies B. (2017). C-type lectins: Their network and roles in pathogen recognition and immunity. Histochem. Cell Biol..

[16] Brown G.D., Willment J.A., Whitehead L. (2018). C-type lectins in immunity and homeostasis. Nat. Rev. Immunol..

[17] Bermejo-Jambrina M., Eder J., Helgers L.C., Hertoghs N., Nijmeijer B.M., Stunnenberg M., Geijtenbeek T.B.H. (2018). C-Type Lectin Receptors in Antiviral Immunity and Viral Escape. Front. Immunol..

[18] Monteiro J.T., Lepenies B. (2017). Myeloid C-Type Lectin Receptors in Viral Recognition and Antiviral Immunity. Viruses.

[19] Van Breedam W., Pöhlmann S., Favoreel H.W., de Groot R.J., Nauwynck H.J. (2014). Bitter-sweet symphony: Glycan–lectin interactions in virus biology. FEMS Microbiol. Rev..

[20] Plassmeyer M.L., Soldan S.S., Stachelek K.M., Roth S.M., Martín-García J., González-Scarano F. (2007). Mutagenesis of the La Crosse Virus glycoprotein supports a role for Gc (1066–1087) as the fusion peptide. Virology.

[21] Shi X., Brauburger K., Elliott R.M. (2005). Role of N-Linked Glycans on Bunyamwera Virus Glycoproteins in Intracellular Trafficking, Protein Folding, and Virus Infectivity. J. Virol..

[22] Hollidge B.S., Nedelsky N.B., Salzano M.-V., Fraser J.W., González-Scarano F., Soldan S.S. (2012). Orthobunyavirus entry into neurons and other mammalian cells occurs via clathrin-mediated endocytosis and requires trafficking into early endosomes. J. Virol..

[23] Hofmann H., Li X., Zhang X., Liu W., Kühl A., Kaup F., Soldan S.S., González-Scarano F., Weber F., He Y. (2013). Severe fever with thrombocytopenia virus glycoproteins are targeted by neutralizing antibodies and can use DC-SIGN as a receptor for pH-dependent entry into human and animal cell lines. J. Virol..

[24] Lozach P.-Y., Kühbacher A., Meier R., Mancini R., Bitto D., Bouloy M., Helenius A. (2011). DC-SIGN as a Receptor for Phleboviruses. Cell Host Microbe.

[25] Teng O., Chen S.-T., Hsu T.-L., Sia S.F., Cole S., Valkenburg S.A., Hsu T.-Y., Zheng J.T., Tu W., Bruzzone R. (2016). CLEC5A-mediated enhancement of the inflammatory response in myeloid cells contributes to influenza pathogenicity in vivo. J. Virol..

[26] Tani H., Shimojima M., Fukushi S., Yoshikawa T., Fukuma A., Taniguchi S., Morikawa S., Saijo M. (2016). Characterization of Glycoprotein-Mediated Entry of Severe Fever with Thrombocytopenia Syndrome Virus. J. Virol..

[27] Mayer S., Moeller R., Monteiro J.T., Ellrott K., Josenhans C., Lepenies B. (2018). C-Type Lectin Receptor (CLR)-Fc Fusion Proteins As Tools to Screen for Novel CLR/Bacteria Interactions: An Exemplary Study on Preselected Campylobacter jejuni Isolates. Front. Immunol..

[28] Cutts T., Grolla A., Jones S., Cook B.W.M., Qiu X., Theriault S.S. (2016). Inactivation of Zaire ebolavirus Variant Makona in Human Serum Samples Analyzed by Enzyme-Linked Immunosorbent Assay. J. Infect. Dis..

[29] Ostrop J., Lang R. (2017). Contact, Collaboration, and Conflict: Signal Integration of Syk-Coupled C-Type Lectin Receptors. J. Immunol..

[30] Kawai T., Akira S. (2008). Toll-like Receptor and RIG-1-like Receptor Signaling. Ann. N. Y. Acad. Sci..

[31] Artigas G., Monteiro J.T., Hinou H., Nishimura S.-I., Lepenies B., Garcia-Martin F. (2017). Glycopeptides as Targets for Dendritic Cells: Exploring MUC1 Glycopeptides Binding Profile toward Macrophage Galactose-Type Lectin (MGL) Orthologs. J. Med. Chem..

[32] Stockinger B., Zal T., Zal A., Gray D. (1996). B cells solicit their own help from T cells. J. Exp. Med..

[33] Tseng Y.-F., Weng T.-C., Lai C.-C., Chen P.-L., Lee M.-S., Hu A.Y.-C. (2018). A fast and efficient purification platform for cell-based influenza viruses by flow-through chromatography. Vaccine.

[34] James K.T., Cooney B., Agopsowicz K., Trevors M.A., Mohamed A., Stoltz D., Hitt M., Shmulevitz M. (2016). Novel High-throughput Approach for Purification of Infectious Virions. Sci. Rep..

[35] Maglinao M., Eriksson M., Schlegel M.K., Zimmermann S., Johannssen T., Götze S., Seeberger P.H., Lepenies B. (2014). A platform to screen for C-type lectin receptor-binding carbohydrates and their potential for cell-specific targeting and immune modulation. J. Control. Release.

[36] Wang H., Nattanmai S., Kramer L.D., Bernard K.A., Tavakoli N.P. (2009). A duplex real-time reverse transcriptase polymerase chain reaction assay for the detection of California serogroup and Cache Valley viruses. Diagn. Microbiol. Infect. Dis..

[37] Li Y.-G., Siripanyaphinyo U., Tumkosit U., Noranate N., A-Nuegoonpipat A., Pan Y., Kameoka M., Kurosu T., Ikuta K., Takeda N. (2012). Poly (I:C), an agonist of toll-like receptor-3, inhibits replication of the Chikungunya virus in BEAS-2B cells. Virol. J..

[38] Murphy F., Gibbs E., Horzinek M., Michael S. (1999). Veterinary Virology.

[39] Gerhauser I., Li L., Li D., Klein S., Elmarabet S.A., Deschl U., Kalkuhl A., Baumgärtner W., Ulrich R., Beineke A. (2018). Dynamic changes and molecular analysis of cell death in the spinal cord of SJL mice infected with the BeAn strain of Theiler’s murine encephalomyelitis virus. Apoptosis.

[40] Kummerfeld M., Meens J., Haas L., Baumgärtner W., Beineke A. (2009). Generation and characterization of a polyclonal antibody for the detection of Theiler’s murine encephalomyelitis virus by light and electron microscopy. J. Virol. Methods.

[41] Chen S.-T., Lin Y.-L., Huang M.-T., Wu M.-F., Cheng S.-C., Lei H.-Y., Lee C.-K., Chiou T.-W., Wong C.-H., Hsieh S.-L. (2008). CLEC5A is critical for dengue-virus-induced lethal disease. Nature.

[42] Van der Vlist M., Geijtenbeek T.B.H. (2010). Langerin functions as an antiviral receptor on Langerhans cells. Immunol. Cell Biol..

[43] Léger P., Tetard M., Youness B., Cordes N., Rouxel R.N., Flamand M., Lozach P.-Y. (2016). Differential Use of the C-Type Lectins L-SIGN and DC-SIGN for Phlebovirus Endocytosis. Traffic.

[44] Pérez de Diego R., Sánchez-Ramón S., López-Collazo E., Martínez-Barricarte R., Cubillos-Zapata C., Ferreira Cerdán A., Casanova J.-L., Puel A. (2015). Genetic errors of the human caspase recruitment domain-B-cell lymphoma 10-mucosa-associated lymphoid tissue lymphoma-translocation gene 1 (CBM) complex: Molecular, immunologic, and clinical heterogeneity. J. Allergy Clin. Immunol..

[45] Yu M., Levine S.J. (2011). Toll-like receptor, RIG-I-like receptors and the NLRP3 inflammasome: Key modulators of innate immune responses to double-stranded RNA viruses. Cytokine Growth Factor Rev..

[46] Tan R.S.T., Ho B., Leung B.P., Ding J.L. (2014). TLR cross-talk confers specificity to innate immunity. Int. Rev. Immunol..

[47] Hefti H.P., Frese M., Landis H., Di Paolo C., Aguzzi A., Haller O., Pavlovic J. (1999). Human MxA Protein Protects Mice Lacking a Functional Alpha/Beta Interferon System against La Crosse Virus and Other Lethal Viral Infections. J. Virol..

[48] Pavlovic J., Schultz J., Hefti H.P., Schuh T., Mölling K. (2000). DNA Vaccination against La Crosse Virus. Intervirology.

[49] Slater L., Bartlett N.W., Haas J.J., Zhu J., Message S.D., Walton R.P., Sykes A., Dahdaleh S., Clarke D.L., Belvisi M.G. (2010). Co-ordinated Role of TLR3, RIG-I and MDA5 in the Innate Response to Rhinovirus in Bronchial Epithelium. PLoS Pathog..

[50] Gibbert K., Dietze K.K., Zelinskyy G., Lang K.S., Barchet W., Kirschning C.J., Dittmer U. (2010). Polyinosinic-Polycytidylic Acid Treatment of Friend Retrovirus-Infected Mice Improves Functional Properties of Virus-Specific T Cells and Prevents Virus-Induced Disease. J. Immunol..

[51] Ngoi S.M., Tovey M.G., Vella A.T. (2008). Targeting Poly(I:C) to the TLR3-Independent Pathway Boosts Effector CD8 T Cell Differentiation through IFN-α/β. J. Immunol..

[52] Luby J.P. (1975). Sensitivities of Neurotropic Arboviruses to Human Interferon. J. Infect. Dis..

[53] Gerhardt R.R., Gottfried K.L., Apperson C.S., Davis B.S., Erwin P.C., Smith A.B., Panella N.A., Powell E.E., Nasci R.S. (2001). First isolation of La Crosse virus from naturally infected Aedes albopictus. Emerg. Infect. Dis..

[54] Harris M.C., Yang F., Jackson D.M., Dotseth E.J., Paulson S.L., Hawley D.M. (2015). La Crosse Virus Field Detection and Vector Competence of Culex Mosquitoes. Am. J. Trop. Med. Hyg..

[55] Sancho D., Reis e Sousa C. (2013). Sensing of cell death by myeloid C-type lectin receptors. Curr. Opin. Immunol..

[56] Hanč P., Fujii T., Iborra S., Yamada Y., Huotari J., Schulz O., Ahrens S., Kjær S., Way M., Sancho D. (2015). Structure of the Complex of F-Actin and DNGR-1, a C-Type Lectin Receptor Involved in Dendritic Cell Cross-Presentation of Dead Cell-Associated Antigens. Immunity.

[57] Neumann K., Castiñeiras-Vilariño M., Höckendorf U., Hannesschläger N., Lemeer S., Kupka D., Meyermann S., Lech M., Anders H.-J., Kuster B. (2014). Clec12a Is an Inhibitory Receptor for Uric Acid Crystals that Regulates Inflammation in Response to Cell Death. Immunity.

[58] Yuita H., Tsuiji M., Tajika Y., Matsumoto Y., Hirano K., Suzuki N., Irimura T. (2005). Retardation of removal of radiation-induced apoptotic cells in developing neural tubes in macrophage galactose-type C-type lectin-1-deficient mouse embryos. Glycobiology.

[59] Yamasaki S., Ishikawa E., Sakuma M., Hara H., Ogata K., Saito T. (2008). Mincle is an ITAM-coupled activating receptor that senses damaged cells. Nat. Immunol..

[60] Kiyotake R., Oh-hora M., Ishikawa E., Miyamoto T., Ishibashi T., Yamasaki S. (2015). Human Mincle Binds to Cholesterol Crystals and Triggers Innate Immune Responses. J. Biol. Chem..

[61] Nagata M., Izumi Y., Ishikawa E., Kiyotake R., Doi R., Iwai S., Omahdi Z., Yamaji T., Miyamoto T., Bamba T. (2017). Intracellular metabolite β-glucosylceramide is an endogenous Mincle ligand possessing immunostimulatory activity. Proc. Natl. Acad. Sci. USA.

[62] Ng W.C., Londrigan S.L., Nasr N., Cunningham A.L., Turville S., Brooks A.G., Reading P.C. (2015). The C-type lectin langerin functions as a receptor for attachment and infectious entry of influenza A virus. J. Virol..

[63] Usami K., Matsuno K., Igarashi M., Denda-Nagai K., Takada A., Irimura T. (2011). Involvement of viral envelope GP2 in Ebola virus entry into cells expressing the macrophage galactose-type C-type lectin. Biochem. Biophys. Res. Commun..

[64] Ng W.C., Liong S., Tate M.D., Irimura T., Denda-Nagai K., Brooks A.G., Londrigan S.L., Reading P.C. (2014). The Macrophage Galactose-Type Lectin Can Function as an Attachment and Entry Receptor for Influenza Virus. J. Virol..

[65] Imai T., Matsumura T., Mayer-Lambertz S., Wells C.A., Ishikawa E., Butcher S.K., Barnett T.C., Walker M.J., Imamura A., Ishida H. (2018). Lipoteichoic acid anchor triggers Mincle to drive protective immunity against invasive group A Streptococcus infection. Proc. Natl. Acad. Sci. USA.

[66] Wevers B.A., Kaptein T.M., Zijlstra-Willems E.M., Theelen B., Boekhout T., Geijtenbeek T.B.H., Gringhuis S.I. (2014). Fungal Engagement of the C-Type Lectin Mincle Suppresses Dectin-1-Induced Antifungal Immunity. Cell Host Microbe.

[67] Lu X., Nagata M., Yamasaki S. (2018). Mincle: 20 years of a versatile sensor of insults. Int. Immunol..

[68] Decout A., Silva-Gomes S., Drocourt D., Blattes E., Rivière M., Prandi J., Larrouy-Maumus G., Caminade A.-M., Hamasur B., Källenius G. (2018). Deciphering the molecular basis of mycobacteria and lipoglycan recognition by the C-type lectin Dectin-2. Sci. Rep..

[69] Goodridge H.S., Reyes C.N., Becker C.A., Katsumoto T.R., Ma J., Wolf A.J., Bose N., Chan A.S.H., Magee A.S., Danielson M.E. (2011). Activation of the innate immune receptor Dectin-1 upon formation of a “phagocytic synapse”. Nature.

[70] Saijo S., Iwakura Y. (2011). Dectin-1 and Dectin-2 in innate immunity against fungi. Int. Immunol..

[71] Yamasaki S., Matsumoto M., Takeuchi O., Matsuzawa T., Ishikawa E., Sakuma M., Tateno H., Uno J., Hirabayashi J., Mikami Y. (2009). C-type lectin Mincle is an activating receptor for pathogenic fungus, Malassezia. Proc. Natl. Acad. Sci. USA.

[72] Ishikawa E., Ishikawa T., Morita Y.S., Toyonaga K., Yamada H., Takeuchi O., Kinoshita T., Akira S., Yoshikai Y., Yamasaki S. (2009). Direct recognition of the mycobacterial glycolipid, trehalose dimycolate, by C-type lectin Mincle. J. Exp. Med..

[73] Poeck H., Bscheider M., Gross O., Finger K., Roth S., Rebsamen M., Hannesschläger N., Schlee M., Rothenfusser S., Barchet W. (2009). Recognition of RNA virus by RIG-I results in activation of CARD9 and inflammasome signaling for interleukin 1β production. Nat. Immunol..

[74] Del Fresno C., Soulat D., Roth S., Blazek K., Udalova I., Sancho D., Ruland J., Ardavín C. (2013). Interferon-β Production via Dectin-1-Syk-IRF5 Signaling in Dendritic Cells Is Crucial for Immunity to C. albicans. Immunity.

[75] Brown C.M., Bidle K.D. (2014). Attenuation of virus production at high multiplicities of infection in Aureococcus anophagefferens. Virology.

[76] Rüdiger D., Kupke S.Y., Laske T., Zmora P., Reichl U. (2019). Multiscale modeling of influenza A virus replication in cell cultures predicts infection dynamics for highly different infection conditions. PLoS Comput. Biol..

[77] Akpinar F., Inankur B., Yin J. (2016). Spatial-Temporal Patterns of Viral Amplification and Interference Initiated by a Single Infected Cell. J. Virol..

[78] Uematsu T., Iizasa E., Kobayashi N., Yoshida H., Hara H. (2015). Loss of CARD9-mediated innate activation attenuates severe influenza pneumonia without compromising host viral immunity. Sci. Rep..

