# Supplementary material for: The CARD9-Associated C-Type Lectin, Mincle, Recognizes La Crosse Virus (LACV) but Plays a Limited Role in Early Antiviral Responses against LACV
Source: Viruses. 2019 Mar 26;11(3):303. doi: 10.3390/v11030303 (PMC6466035; doi:10.3390/v11030303)
Supplement: Supplementary file 1 [file viruses-11-00303-s001.pdf]

Supplementary Materials

The CARD9-associated C-type lectin, Mincle, recognizes La Crosse virus (LACV) but plays a limited role in early antiviral responses against LACV

João T. Monteiro [1], Kathleen Schön [1,2], Tim Ebbecke [1], Ralph Goethe [3], Jürgen Ruland [4,5,6,7], Wolfgang Baumgärtner [8], Stefanie C. Becker [2,*] and Bernd Lepenies [1,*]

1 Immunology Unit & Research Center for Emerging Infections and Zoonoses, University of Veterinary Medicine Hannover, Hannover, Germany.

2 Institute for Parasitology and & Research Center for Emerging Infections and Zoonoses, University of Veterinary Medicine Hannover, Hannover, Germany.

3 Institute for Microbiology, University of Veterinary Medicine Hannover, Hannover, Germany.

4 Institute of Clinical Chemistry and Pathobiochemistry, School of Medicine, Technical University of Munich, Munich, Germany.

5 Department of Pathology, University of Veterinary Medicine Hannover, Hannover, Germany.

* Correspondence: stefanie.becker@tiho-hannover.de; bernd.lepenies@tiho-hannover.de

Table S1 – Primers used to amplify the extracellular domain of murine or human C-type lectins.

CLR	Primers
Murine CLEC12B	FW 5' CCATGGACTTTCTCCTAGGATGTCTG 3'
	RV 5' AGATCTGCATGGGTTTGCAATAGGTC 3'
Murine CLEC13A	FW 5' GAATTCCTGTCCTTCATCTACCTGGGTCC 3'
	RV 5' CCATGGTGTCATATGGGATTGCTGCTTT3'
Murine CLEC5A	FW 5' GAATTCCCCCACGGAGAGCTACGGAACCA 3'
	RV 5' CCATGGTGGCATTCATTTCGCAGATCCA 3'
Murine Langerin	FW 5' GATATCAGGTCGTGTGGACGATGCTGAGGT 3'
	RV 5' CCATGGTTTGGACGTAGGGCCTCTTGCAG 3'
Murine Dectin-1	FW 5' GAATTCTTCAGGGAGAAATCCAGAGG 3'
	RV 5' AGATCTTGAAGAAGTATTGCAGATTTGGTT 3'
Murine Dectin-2	FW 5' GAATTCCTGGAGCACCAGTGAGCAGAAC 3'
	RV 5' CCATGGAGAAAACATCATTCCAGCCCC 3'
Murine Mincle	FW 5' CCATGGGGCAGAACTTACAGCCACAT 3'
	RV 5' AGATCTGTCCAGAGGACTTATTTCTG 3'
Murine DCAR	FW 5' CCATGGAACTTGACAGGTACCATTCATT 3'
	RV 5' AGATCTTAAGTTTATTTTCTTCATCTGAC 3'
Murine SIGNR3	FW 5' GAATTCCATGCAACTGAAGGCTGAAG 3'
	RV 5' AGATCTTTTGGTGGTGCATGATGAGG 3'
Murine MGL-1	FW 5' CCAGTTAAGGAGGGACCTAGGCAC 3'
	RV 5' AGCTCTCCTTGGCCAGCTTCATC 3'
Human DC-SIGN	FW 5' GAATTCGTCCAAGGTCCCCAGCTCCAT 3'
	RV 5' CCATGGACGCAGGAGGGGGGTTTGGGGT 3'
Human L-SIGN	FW 5' GAATTCCTATCAAGAACTGACCGATTTG 3'
	RV 5' CCATGGATTCGTCTCTGAAGCAGGC 3'

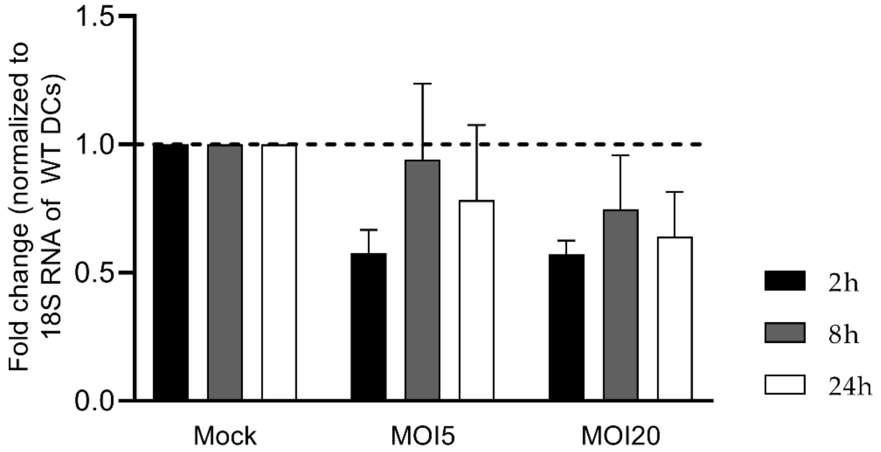

Figure S1 – CARD9 mRNA expression levels in LACV-infected WT DCs. The mock-infected DCs were used as a baseline at the respective time points to evaluate differences in CARD9 expression. Data shown are mean ± SEM; three independent experiments were performed. A two-way ANOVA with a Tukey´s honest significance test was used to compare differences between the different groups and $p < 0.05$ was considered significant (* $p < 0.05$).

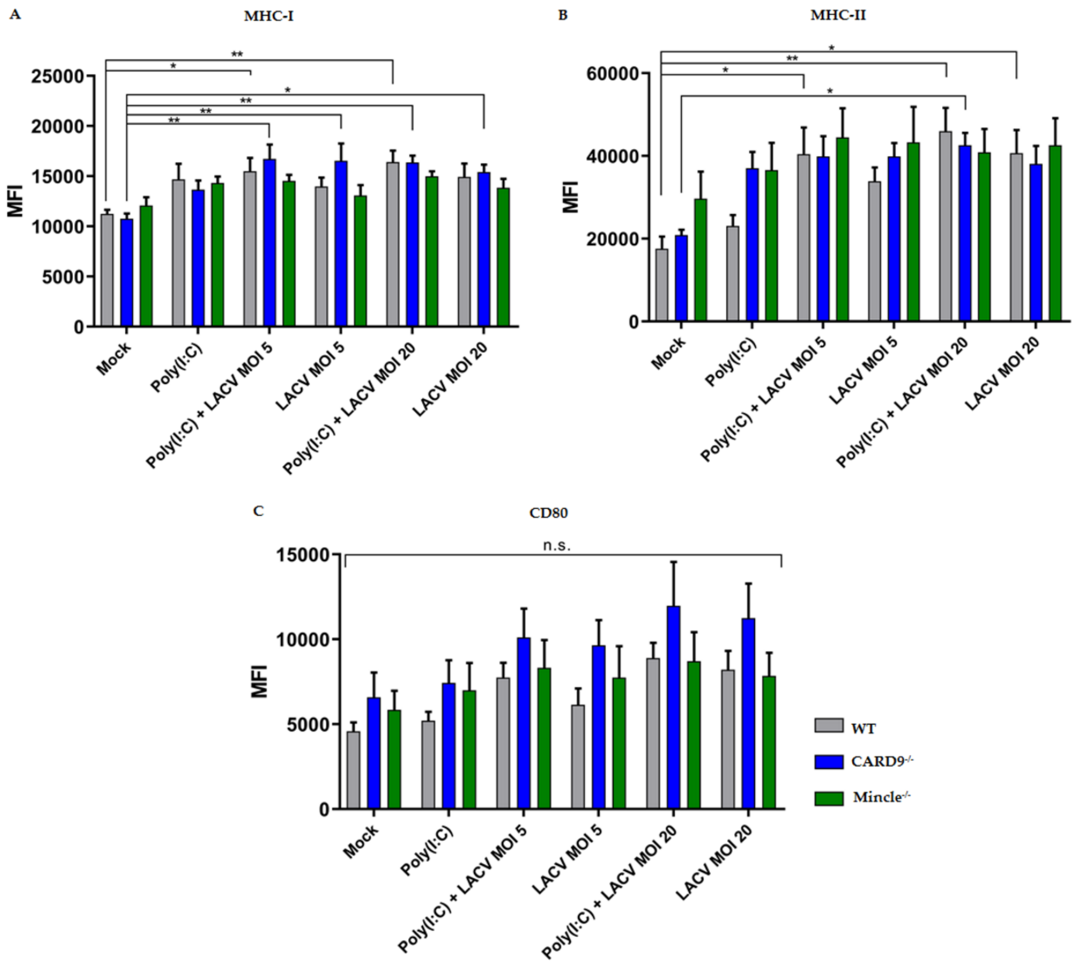

Figure S2 – Effector functions of Mincle$^{-/-}$, CARD9$^{-/-}$ and WT DCs after 8h of LACV infection. **(A)** Surface expression of MHC-I; **(B)** Surface expression of MHC-II; **(C)** Surface expression of CD80 in Mincle$^{-/-}$, CARD9$^{-/-}$ or WT DCs. Data represented are mean ± SEM (of three independent experiments. Data are presented as MFI (median fluorescence intensity) values. A two-way ANOVA with a Tukey´s honest significance test was performed and $p < 0.05$ was considered significant (* $p < 0.05$, ** $p < 0.01$, *** $p < 0.001$, **** $p < 0.0001$).

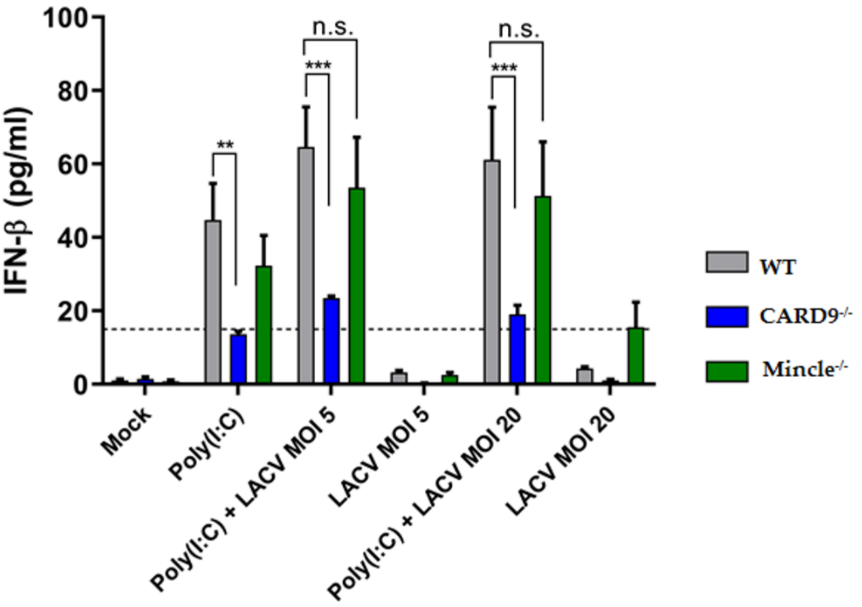

Figure S3 – CARD9$^{-/-}$ DCs exhibit a decreased IFN-β production. IFN-β production was evaluated in Mincle$^{-/-}$, CARD9$^{-/-}$ and WT DCs after 24h of stimulation with LACV. Data represented are mean ± SEM of four independent experiments. The dotted line represents the limit of detection of the ELISA kit (16 pg/ml). A two-way ANOVA with a Tukey´s honest significance test was performed and a $p < 0.05$ was considered significant (* $p < 0.05$, ** $p < 0.01$, *** $p < 0.001$).

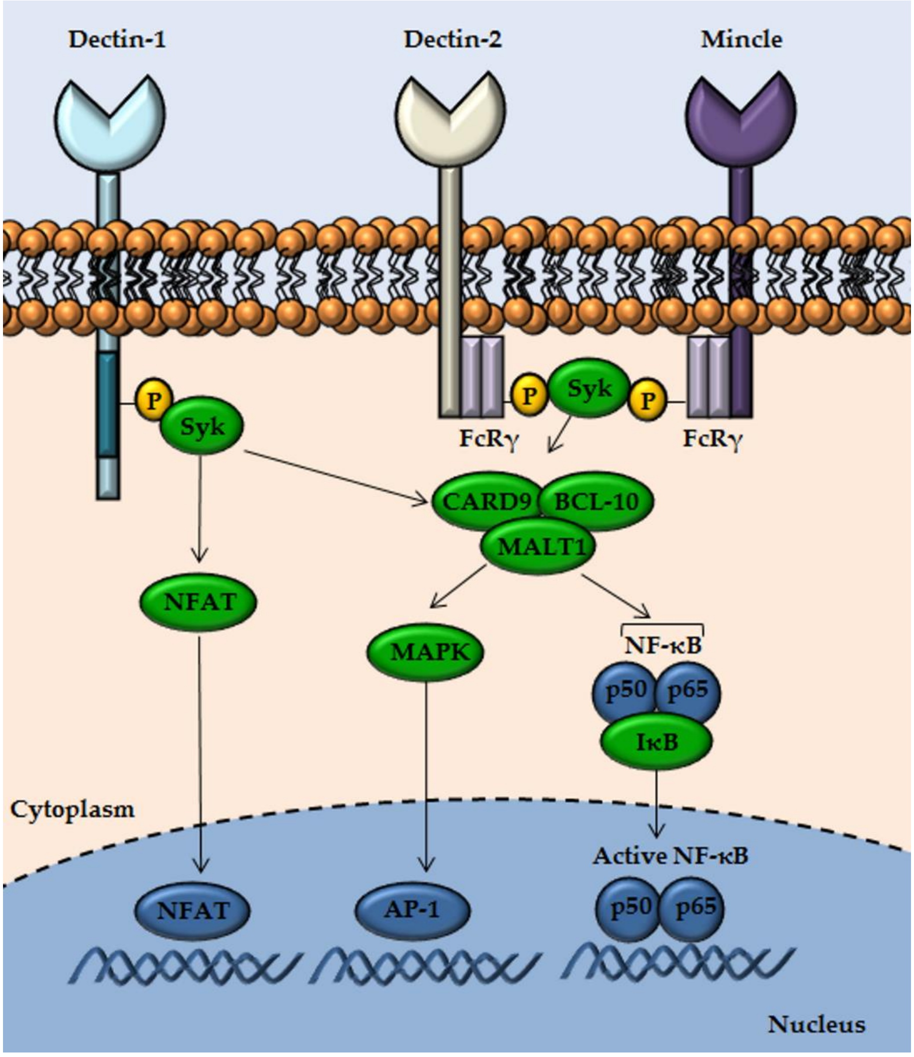

Figure S4 – Engagement of CARD9-associated CLRs and downstream signaling cascades. CLRs recognize conserved glycans on the surface of pathogens or host cells. CLR engagement prompts the use of the adaptor chain Fc receptor γ-chain (FcRγ) for Dectin-2 and Mincle mediated activation of spleen tyrosine kinase, Syk; while Dectin-1 only requires the phosphorylation of its intracellular hemi-immunoreceptor tyrosine-based activating motif (hemITAM). Activation of Syk leads to assembly of the caspase-recruitment domain 9 (CARD9)/B cell lymphoma/leukaemia 10 (BCL10)/mucosa-associated lymphoid tissue lymphoma translocation protein 1 (Malt1) (CARD9/BCL10/Malt1) complex and/or further activation of the transcription factors nuclear factor of activated T cell (NFAT), nuclear factor-κB (NF-κB) and activator protein-1 (AP-1), by mitogen-activated protein kinase (MAPK signaling). Activation of these transcription factors is required for early pro-inflammatory responses.
